# Hypertension in HIV-Infected Patients Receiving Antiretroviral Therapy in Northeast Ethiopia

**DOI:** 10.1155/2019/4103604

**Published:** 2019-12-23

**Authors:** Temesgen Fiseha, Alemu Gedefie Belete, Henok Dereje, Abebe Dires

**Affiliations:** ^1^Department of Clinical Laboratory Science, College of Medicine and Health Sciences, Wollo University, Dessie, Ethiopia; ^2^Department of Nursing, College of Medicine and Health Sciences, Wollo University, Dessie, Ethiopia

## Abstract

**Background:**

With prolonged survival and aging of persons with HIV on combination antiretroviral therapy (ART), hypertension has emerged as a significant cause of morbidity and mortality globally. However, little is known about the burden of this comorbid condition among adults living with HIV in sub-Saharan Africa. In this study, we aimed to determine the prevalence and factors associated with hypertension among HIV-infected patients receiving ART in Northeast Ethiopia.

**Methods:**

A cross-sectional study was conducted at the ART clinic of Dessie Referral Hospital, Northeast Ethiopia, between January and May 2018. HIV-infected patients who were on ART for at least 12 months were included in the study. Demographic, clinical, and laboratory data were collected from each participant. Hypertension was defined as a systolic blood pressure (BP) of ≥140 mmHg and/or diastolic BP of ≥90 mmHg or a reported use of antihypertensive medication. Univariable and multivariate analyses were performed to identify factors associated with hypertension.

**Results:**

A total of 408 patients were studied with a mean (±SD) age of 37 ± 10.3 years, and 66.9% were female. The prevalence of hypertension was 29.7% (95% CI, 25.3–35.0%). Nearly 75% of the patients with hypertension were previously undiagnosed. In a univariate analysis, older age, male gender, a family history of hypertension, duration of HIV infection, duration on ART, high body mass index, low CD4 count, diabetes, and renal impairment were associated with hypertension. Multivariate analysis revealed older age (AOR = 2.08; 95% CI, 1.13–3.83), male gender (AOR = 1.64; 95% CI, 1.01–2.65), longer duration on ART (AOR = 1.91; 95% CI, 1.14–3.20), high body mass index (AOR = 3.32; 95% CI, 1.13–9.77), and diabetes (AOR = 2.76; 95% CI, 1.29–5.89) as independent risk factors of hypertension.

**Conclusions:**

Hypertension is highly prevalent among HIV-infected patients on ART attending our clinic in Northeast Ethiopia but is mostly undiagnosed. These findings highlight the need for integrating hypertension management into routine HIV care to prevent adverse outcomes and improve health of people living with HIV on ART.

## 1. Introduction

The widespread introduction of combined active antiretroviral therapy (ART) for human immunodeficiency virus (HIV) infection has dramatically reduced AIDS-related mortality and increased life expectancy among people living with HIV in both high- and low-income countries [1–3]. With prolonged survival and aging of persons with HIV, chronic comorbidities, including hypertension, have emerged as significant causes of morbidity and mortality among these patients with access to ART [4, 5]. Hypertension is now recognized as an important chronic comorbid condition occurring in people living with HIV and is associated with increased morbidity and mortality [6, 7]. In patients with HIV, hypertension has been shown to be associated with an increased risk for hospitalization and adverse cardiovascular and renal outcomes, including progression to end-stage renal disease that consequently reduces life expectancy and add to the already high costs of treatment for this population [8–10].

People living with HIV on combination ART are at an increased risk for hypertension than HIV-uninfected individuals [6, 11]. Data from around the globe demonstrated that 35% of all HIV-infected adults on ART have hypertension, compared with an estimated 30% of HIV-uninfected adults [12]. Many risk factors could contribute to the higher prevalence of hypertension in such HIV-infected population, including older age, male gender, family history of hypertension, longer duration of HIV infection, low CD4 count, high viral burden, and high body mass index [7, 13]. In addition, the use of certain medications included in combination ART regimens have been also shown to contribute to the increased risk of hypertension in people living with HIV [14, 15]. In this regard, a recent meta-analysis of more than 44,000 HIV-infected patients showed the risk of hypertension to be as two times higher in ART-exposed patients compared with treatment-naive patients [16].

With the new “test and treat” policy of the WHO [17], it is obvious that the number of ART-exposed patients will grow exponentially, with consequential increase in the burden of hypertension. However, limited data are available regarding the burden of hypertension among adults living with HIV on ART in sub-Saharan Africa, where more than 70% of those living with HIV reside [18]. Therefore, in this study, we aimed to determine the prevalence and factors associated with hypertension among HIV-infected adults receiving ART in Northeast Ethiopia.

## 2. Methods

### 2.1. Study Settings and Participants

Detailed methods for the study have been published previously [19]. In brief, an instruction-based cross-sectional study was conducted from January to May 2018 at the ART clinic of Dessie Referral Hospital (DRH), Northeast Ethiopia. DRH is found in Dessie town of Amhara regional state, which is located 401 km northeast of the capital Addis Ababa, Ethiopia. The hospital serves as a referral center for the Wollo and surrounding zones and provides HIV/AIDS interventions including free diagnosis, treatment, and monitoring. At the time our study was conducted, criteria for ART initiation was treat all regardless of CD4 cell count or clinical stage. HIV-infected adults (aged > 18 years) attending the ART outpatient clinic for regular follow-up, who were on ART for at least 12 months and willing to provide consent were included in the study. Patients were excluded if they were pregnant, hospitalized, and/or with critical illness that rendered them unable to answer the questionnaires.

### 2.2. Sample Size Determination

The sample size was determined by considering 50% prevalence rate of hypertension. Using single population proportion formula, with the following parameters: *Z* = the level of statistical significance with a 95% confidence interval (CI) of 1.96, and a precision level of 0.05, then the minimum sample size obtained was 384. After adding 10% to account for nonrespondents, a total of 422 HIV-infected individuals on combination ART were consecutively included in the study. Fourteen patients with missing clinical data were excluded, leaving 408 patients for the final analysis.

### 2.3. Data Collection and Laboratory Measurements

Eligible patients were approached and invited to participate while they were attending the ART clinic for their regular follow-up. For consenting patients, structured questionnaires were used to collect data on the sociodemographic, medical and family history, and behavioral (i.e., smoking and alcohol use) factors. Anthropometric data such as weight (in kilograms), height (in meters), and blood pressure (in mm Hg) were also measured. Body weight and height were measured in light clothes without footwear, and the body mass index (BMI) was calculated as weight divided by height squared (kg/m^2^). Blood pressure was measured in the right arm three times at an interval of at least 5 min using a manual sphygmomanometer, with the patient remaining in a sitting position for at least 15 min before the first measurement. The average of the three measurements was recorded.

Clinical measures, including duration of HIV infection (i.e., duration since HIV diagnosis), duration on ART, and types of ART regimens, were abstracted from patients' database. Blood samples were collected in the early morning after an overnight fasting. Fasting plasma glucose was measured by enzymatic GOD-PAP method as mg/dl using A25 Biosystems clinical chemistry analyzer (BioSystems S.A., Barcelona, Spain). Serum creatinine was measured using Jaffe kinetic method (as mg/dl) for estimating glomerular filtration rate (eGFR) according to the Modification of Diet in Renal Disease (MDRD) study equation [20]. CD4 cell count was measured using the BD FACSCOUNT system (Becton Dickenson and Company, California, USA).

### 2.4. Definitions

Hypertension was defined as a self-reported use of antihypertensive medication and/or systolic blood pressure of ≥140 mmHg and/or diastolic blood pressure of ≥90 mmHg on at least two occasions. Diabetes mellitus was defined as fasting plasma glucose level of ≥126 mg/dl on two consecutive study visits [21] or a previous diagnosis of diabetes (on treatment for insulin injection/oral medication treatment). Participants were categorized as smokers, if they smoke at least one cigarette for the last 12 months, and alcohol consumer, if they consume at least twice weekly of any alcoholic drinks. BMI was classified as normal/underweight (BMI <25 kg/m^2^) and overweight/obese (BMI ≥25 kg/m^2^).

### 2.5. Statistical Analysis

Data were entered into “EpiData version 3.1” and exported to SPSS version 20.0 statistical software for analysis. Data were reported using mean ± standard deviation (SD) for continuous variables and proportions for categorical variables. The chi-square (*χ*^2^) test was used to compare categorical variables, whereas the *t*-test was used to compare continuous variables. Multivariate stepwise logistic regression analysis with backward selection of variables with univariate *P* values of <0.25 was conducted to identify factors independently associated with hypertension in the study population. Odds ratios (OR) and 95% confidence intervals (95% CI) were also obtained. A *P* value of <0.05 was used to indicate statistical significance.

## 3. Results

### 3.1. Demographic and Clinical Characteristics of the Study Participants

A total of 408 patients were included in the analysis (66.9% female). Demographic and clinical characteristics are shown in Table 1. The mean age (SD) was 37 (10.3) years overall, 39 (11.5) years in men, and 36 (9.5) years in women (*P*=0.002). All patients were on combination ART, and overall 150 (36.8%) were on AZT regimen. Sixteen participants (3.9%) were current smokers, and 62 (15.2%) alcohol users, with significant sex differences (both *P* < 0.01). Men compared with women were more likely to have a family history of hypertension (*P*=0.048). Mean (SD) body mass index was 20.4 (2.1) kg/m^2^, with no difference between men and women (*P*=0.233). The mean (SD) systolic and diastolic blood pressures did not differ between men and women (both *P* > 0.05). Overall, the proportion of participants with diabetes mellitus was 8.8% and 9.8%, respectively, and had moderate to severe renal impairment (eGFR < 60 ml/min/1.73 m^2^), with significant difference between men and women (*P*=0.021 and *P* < 0.001, respectively).

### 3.2. Prevalence of Hypertension

The prevalence of hypertension was 29.7% (95% CI: 25.3–35.0%). Of the 121 patients with hypertension, 31 (25.6%) were already on pharmacotherapy and 90 (74.4%) were newly diagnosed. The prevalence of hypertension was significantly higher in older patients (older than 45 years: 53.8%) than in their younger counterparts (23.9%; *P* < 0.001). Higher prevalence of hypertension was also observed in men than in women and in those with longer duration of HIV infection, longer duration of ART exposure, a family history of hypertension, high BMI, low CD4 count, diabetes mellitus, or impaired renal function (low eGFR). No significant difference was found in hypertension prevalence for the other characteristics recorded (Table 2).

### 3.3. Factors Associated with Hypertension

In multivariate analysis, older age (AOR = 2.08; 95% CI: 1.13–3.83; *P*=0.019), male sex (AOR = 1.64; 95% CI: 1.01–2.65; *P*=0.044), longer duration on ART (AOR = 1.91; 95% CI: 1.14–3.20; *P*=0.014), high BMI (BMI above or equal to 25 kg/m^2^: AOR = 3.32; 95% CI: 1.13–9.77; *P*=0.032), and diabetes mellitus (AOR = 2.76; 95% CI: 1.29–5.89; *P*=0.009) were independently associated with an increased risk of hypertension (Table 3).

## 4. Discussion

Our study found that the prevalence of hypertension among HIV-infected adults on ART in our setting was high (29.7%), and it was previously undiagnosed in about three quarters (74.4%). The overall prevalence of hypertension in this study was comparable to 34% prevalence reported from Southwest Ethiopia [22] but higher than that reported from studies conducted in other parts of the country, including 12.7% in Eastern Ethiopia [23], 15.9% in Southern Ethiopia [24] and 17.1% in Northwest Ethiopia [25]. Our prevalence estimate of hypertension was also comparable to studies conducted in Tanzania (28.7%) [26], Senegal (28.1%) [27], and Uganda (27.9%) [28]. The estimated prevalence of hypertension in our study population was higher than the 19.1% prevalence reported in the South African study [29] and 19.5% and 23.7% in two Malawi studies [30, 31] but lower than 38% reported in the Cameroon study [32] and 38.6% in recent South African study [33]. The observed difference could be due to variation in the duration and stage of HIV infection, and the types of and duration of ART exposure or due to variation in the lifestyle and age distribution of the studied subjects. The difference could also be a consequence of differences in study design, the definition of hypertension, and the range of demographic and metabolic-related factors.

The increased risk of hypertension observed among older HIV-infected patients on ART in our study is consistent with previous studies [14, 24, 27, 31, 34]. These and other related studies [15, 35] showed that older patients had an increased risk of prevalent hypertension and should be targeted for frequent blood pressure monitoring and the identification of other risk factors to encourage lifestyle modification. We also observed a significant association between male gender and hypertension in our study population, and this was in agreement with previous reports [15, 34, 35], which indicated that male patients were at an increased risk of hypertension during their time on ART. However, no significant association between gender and hypertension was observed in a previous study conducted in the country [23]. A family history of hypertension was also significantly associated with prevalent hypertension only in univariate analyses but not in multivariate analyses. This was consistent with other study finding [9]. In contrast to this, a previous study showed that HIV-infected patients with a family history of hypertension were more vulnerable to developing hypertension when on ART [11]. In that study a family history of hypertension was reported in 39.0% of the HIV population. However, the percentage of a family history of hypertension in our study population was low (13.7% of the total), which may explain why a family history of hypertension is unrelated to prevalent hypertension in our study. It is possible that a low prevalence of self-reported family history of hypertension may be related to participants not being aware of hypertension in their family members.

The multivariate analysis of the present study however found an independent association between duration of ART exposure and the presence of hypertension, and this is consistent with other related studies [9, 14, 23, 34]. In that study, Baekken et al. found the highest risk of prevalent hypertension in those treated with combination ART for more than 5 years (44.4%) [34]. Another study also found an increased risk of hypertension in those with long-term exposure to ART [36]. This was also supported by a recent meta-analysis of HIV-infected patients, which showed that the duration of ART exposure was associated with increased blood pressure and hypertension risk [16]. The cumulative duration of antiretroviral treatment effect may be mediated through the increasing age and the age-related increase in comorbidities, such as weight gain and ART-related changes in body composition, or it could be mediated directly through changes in endothelial function [7, 13].

Higher BMI was found to be associated with an increased risk of prevalent hypertension in our patients. This finding was in agreement with the study from the country [23] and other studies elsewhere [15, 27, 31, 35], indicating that HIV-infected patients classified as overweight or obese were at an increased risk of developing hypertension. Our study also found a significant association between the presence of diabetes and prevalence of hypertension. This is also supported by a previous study conducted in the country [23], which showed that raised blood glucose was significantly associated with prevalent hypertension. Additionally, previous study has shown that diabetes was also an independent predictor of hypertension among HIV-infected adults in the ART era [35]. In this study, renal insufficiency was also significantly associated with an increased risk of hypertension only in univariate analyses but not in multivariate analyses. An independent association between renal insufficiency and hypertension in patients living with HIV on ART has previously been reported in other related studies [35, 37].

Limitations of the present study are mainly related to the observational design and cross-sectional nature of the current analyses. The results reported herein are only associations from which no conclusions regarding causality can be drawn. Given the drugs were studied in combinations, we were unable to assess the association between exposure to each of the individual ARV drugs and the risk of hypertension. On the other hand, information on other environmental factors, such as physical activity or diet and salt intake, was not collected. The stratification of renal function has been made based on the MDRD-4 formula, the validation of which is lacking among Ethiopian adults. Finally, our study reports data from large, public-sector government ART clinic, so our results may not be generalizable to the overall population.

## 5. Conclusions

In conclusion, hypertension was highly prevalent among HIV-infected adults on ART attending our clinic in Northeast Ethiopia, but it was mostly undiagnosed. Older age, male gender, ART duration, high BMI, and comorbid diabetes were independently associated with hypertension. These findings highlight the need for integrating hypertension screening, diagnosis, and treatment into routine HIV care to prevent disease-related adverse outcomes and improve health of people living with HIV on ART.

## Figures and Tables

**Table 1:** Demographic and clinical characteristics of study participants by sex.

Characteristics	Overall (*N* = 408)	Men (*N* = 135)	Women (*N* = 273)	*P* value
Mean age (SD)	37 (10.3)	39 (11.5)	36 (9.5)	0.002
Age group in years, *n* (%)				
18–34	169 (41.4)	43 (31.9)	126 (46.2)	0.001
35–45	161 (39.5)	51 (37.8)	110 (40.2)	
>45	78 (19.1)	41 (30.4)	37 (13.6)	
Residence, *n* (%)				
Rural	88 (21.6)	38 (28.1)	50 (18.3)	0.023
Urban	320 (78.4)	97 (71.9)	223 (69.7)	
Education, *n* (%)				
<High school	240 (58.8)	67 (49.6)	173 (63.4)	0.008
≥High school	168 (41.2)	68 (50.4)	100 (36.6)	
Duration of HIV infection, *n* (%)				
≤5 years	144 (35.3)	41 (30.4)	103 (37.7)	0.143
>5 years	264 (64.7)	94 (69.6)	170 (62.3)	
Duration on ART, *n* (%)				
≤5 years	227 (55.6)	67 (49.6)	160 (58.6)	0.086
>5 years	181 (44.4)	68 (50.4)	113 (41.4)	
ART regimen				
AZT + 3TC + EFV	66 (16.2)	36 (26.7)	30 (11.0)	0.001
TDF + 3TC + NVP	71 (17.4)	9 (6.7)	62 (22.7)	
AZT + 3TC + NVP	84 (20.6)	16 (11.9)	68 (24.9)	
TDF + 3TC + EFV	76 (18.6)	28 (20.7)	48 (17.6)	
Other	111 (27.2)	46 (31.4)	65 (23.8)	
Current smoking, *n* (%)	16 (3.9)	11 (8.1)	5 (1.8)	0.002
Alcohol use, *n* (%)	62 (15.2)	45 (33.3)	17 (6.2)	0.001
Family history of hypertension, *n* (%)				
Yes	56 (13.7)	25 (18.5)	31 (11.4)	0.048
No	352 (86.3)	110 (81.5)	242 (88.6)	
Body mass index, kg/m^2^, mean (SD)	20.4 (2.1)	20.2 (2.3)	20.5 (2.0)	0.233
Body mass index ≥25 kg/m^2^, *n* (%)	13 (3.2)	4 (3.0)	9 (3.3)	0.857
WHO clinical stage I/II, *n* (%)	329 (80.6)	120 (88.9)	209 (76.6)	0.003
CD4 count (cells/mm^3^), mean (SD)	343.1 (311.6)	340.7 (326.6)	344.3 (304.5)	0.912
Blood pressure (BP) (mmHg), mean (SD)				
Systolic BP	127.2 (12.8)	128.1 (13.5)	126.8 (12.5)	0.336
Diastolic BP	82.0 (6.0)	82.2 (5.0)	81.9 (6.4)	0.682
Fasting plasma glucose (mg/dl), mean (SD)	105.7 (39.0)	113.1 (49.7)	102.1 (31.9)	0.007
Diabetes mellitus, *n* (%)	36 (8.8)	18 (13.3)	18 (6.6)	0.021
GFR (ml/min/1.73 m^2^), mean (SD)	116.0 (43.8)	132.2 (46.7)	108.3 (40.0)	0.001

WHO clinical stage I/II: I (asymptomatic or persistent generalized lymphadenopathy) or II (symptomatic, not categories I or II); current smoking: smoke at least one cigarette for the last 12 months; alcohol use: consume at least twice weekly of any alcoholic drinks. CD_4_: cluster of differentiation, GFR: glomerular filtration rate, SD: standard deviation.

**Table 2:** Prevalence of hypertension among HIV-infected patients on ART at DRH in Northeast Ethiopia.

Characteristics	Hypertension status		*P* value
	Yes (*N* = 121), *n* (%)	No (*N* = 287), *n* (%)	
Age (years)			<0.001
≤45	79 (23.9)	251 (76.1)	
>45 years	42 (53.8)	36 (46.2)	
Sex			0.003
Male	53 (39.3)	82 (60.7)	
Female	68 (24.9)	205 (75.1)	
Residence			0.772
Rural	25 (28.4)	63 (71.6)	
Urban	96 (30.0)	224 (70.0)	
Education			0.484
<High school	68 (28.3)	172 (71.7)	
≥High school	53 (31.5)	115 (68.5)	
Duration of HIV infection (years)			0.041
≤5	34 (23.4)	111 (76.6)	
>5	87 (33.1)	176 (66.9)	
Duration on ART (years)			<0.001
≤5	47 (20.7)	180 (79.3)	
>5	74 (40.9)	107 (59.1)	
ART regimen			0.911
AZT + 3TC + EFV	17 (25.8)	49 (74.2)	
TDF + 3TC + NVP	20 (28.2)	51 (70.2)	
AZT + 3TC + NVP	25 (29.8)	59 (70.2)	
TDF + 3TC + EFV	25 (32.9)	51 (67.1)	
Other	34 (30.6)	77 (69.4)	
Smoking			0.887
Yes	5 (31.2)	11 (68.8)	
No	116 (29.6)	276 (70.4)	
Alcohol use			0.164
Yes	23 (37.1)	39 (62.9)	
No	98 (28.3)	248 (71.7)	
Family history of hypertension			0.044
Yes	23 (41.1)	33 (58.9)	
No	98 (27.8)	254 (72.2)	
Body mass index (kg/m^2^)			0.011
<25	113 (28.6)	282 (71.4)	
≥25	8 (61.5)	5 (38.5)	
WHO clinical stage			0.876
Early stage (I and II)	97 (29.5)	232 (70.5)	
Advanced stage (III and IV)	24 (30.4)	55 (69.5)	
CD4 count (cells/mm^3^)			0.020
<350	86 (33.7)	169 (66.3)	
≥350	35 (22.9)	118 (77.1)	
Diabetes mellitus			<0.001
Yes	21 (58.3)	15 (41.7)	
No	100 (26.9)	272 (73.1)	
Glomerular filtration rate (ml/min/1.73 m^2^)			0.005
<60	18 (45.0)	22 (55.0)	
60–89.9	27 (39.7)	41 (60.3)	
≥90	76 (25.3)	224 (74.7)	

**Table 3:** Factors associated with hypertension among HIV-infected patients on ART at DRH in Northeast Ethiopia.

Characteristics	Crude OR (95% CI)	Adjusted OR (95% CI)
Age (years)		
≤45	1	1
>45	3.71 (2.22–6.19)	2.08 (1.13–3.83)
Sex		
Female	1	1
Male	2.00 (1.25–3.57)	1.64 (1.01–2.65)
Duration of infection (years)		
≤5	1	1
>5	1.61 (1.02–2.56)	0.92 (0.54–1.58)
Duration on ART (years)		
≤5	1	1
>5	2.67 (1.71–4.14)	1.91 (1.14–3.20)
CD4 cell count (cells/mm^3^)		
≥350	1	1
<350	1.72 (1.09–2.71)	1.83 (0.97–3.46)
Body mass index (kg/m^2^)		
<25	1	1
≥25	3.99 (1.28–12.47)	3.32 (1.13–9.77)
Family history of hypertension		
No	1	1
Yes	1.81 (1.01–3.23)	0.96 (0.62–2.74)
Diabetes mellitus		
No	1	1
Yes	3.81 (1.89–7.68)	2.76 (1.29–5.89)
Glomerular filtration rate (ml/min/1.73 m^2^)		
≥90	1	1
60–89.9	1.94 (1.12–3.37)	1.57 (0.83–2.95)
<60	2.41 (1.23–4.74)	1.80 (0.81–4.00)

## Data Availability

The data of this study cannot be shared publicly due to the presence of sensitive (confidential) participants' information and additional data than those used in this publication. But the data are available from the corresponding author on reasonable request.

## Abbreviations

AOR: Adjusted odds ratio
ART: Antiretroviral therapy
BMI: Body mass index
CI: Confidence interval
DRH: Dessie referral hospital
eGFR: Estimated glomerular filtration rate
HCV: Hepatitis C virus
HIV: Human immunodeficiency virus.
